# Desflurane post-conditioning inhibits HMGB1 nucleocytoplasmic translocation via the SIRT1/HMGB1 axis to attenuate hepatic ischemia-reperfusion-induced acute lung injury in young rats

**DOI:** 10.3389/fphar.2026.1847195

**Published:** 2026-06-29

**Authors:** Yimei Cao, Yingli Cao, Hongyin Du, Hengchang Ren, Xiangming Che

**Affiliations:** 1 Department of Anesthesiology, Beijing Obstetrics and Gynecology Hospital, Capital Medical University, Beijing Maternal and Child Health Care Hospital, Beijing, China; 2 First Central Hospital of Tianjin Medical University, Tianjin, China; 3 Department of Anesthesiology, Peking University Third Hospital, Beijing, China; 4 Department of Anesthesiology, Tianjin First Central Hospital, Tianjin, China

**Keywords:** acute lung injury, desflurane, hepatic ischemia-reperfusion, high mobility group protein B1, silent information regulator 2 related enzyme 1

## Abstract

Acute lung injury (ALI) is a common clinical complication in pediatric patients undergoing perioperative liver transplantation, and therapeutic approaches are limited. While desflurane exhibits organ-protective potential, its specific efficacy against hepatic ischemia-reperfusion (HIR)-induced ALI in the developing lung, as well as the underlying molecular mechanisms, remain poorly understood. This study aimed to investigate the protective effects of desflurane and delineate the regulatory pathways involved. 2 week-old Sprague-Dawley rats were subjected to 70% warm HIR to simulate pediatric liver transplantation. Desflurane was administered at the onset of reperfusion. The severity of lung injury, inflammatory responses, and relevant intracellular signaling pathways were evaluated using histological and molecular analyses. *In vitro*, NR8383 alveolar macrophages underwent oxygen-glucose deprivation (OGD) reoxygenation to further verify the underlying mechanisms. The results showed that desflurane post-conditioning significantly improved ALI induced by HIR, characterized by reduced acute lung injury scores and inhibited release of albumin, tumor necrosis factor (TNF)-α, macrophage inflammatory protein (MIP) −2 in bronchoalveolar lavage fluid, and HMGB1 in serum. Furthermore, we found that desflurane could upregulate the silent information regulator 2-related enzyme (SIRT) 1 level and inhibit the nucleocytoplasmic translocation and acetylation of HMGB1 and its related signaling pathways. Desflurane upregulated SIRT1 expression, which directly mediated the deacetylation of HMGB1 at the K29/K177 residues *in vitro*. Notably, the above-mentioned effects of desflurane on ALI were markedly attenuated by Ex527, a specific inhibitor of SIRT1, both *in vivo* and *in vitro*. Our data indicate that desflurane post-conditioning attenuates HIR-induced lung injury in young rats by restoring SIRT1-mediated HMGB1 deacetylation and provides a new research direction for the clinical development of safe and effective perioperative ALI prevention and treatment strategies in pediatric liver transplantation.

## 1 Introduction

Pediatric liver transplantation has increased markedly worldwide. Although survival rates among pediatric patients have improved significantly, remote organ injury caused by hepatic ischemia-reperfusion (HIR) injury continues to compromise their postoperative quality of life (Hassan et al., 2025). As an unavoidable process during liver transplantation, HIR involves a complex cascade, including microcirculatory dysfunction, hypoxia, oxidative stress, activation of innate and adaptive immune responses, and cell death (Liu and Man, 2023). In pediatric patients, HIR-induced acute lung injury (ALI) and its most serious form, acute respiratory distress syndrome (ARDS), are critical complications during the perioperative period of liver transplantation (Kohli et al., 2018). Currently, there are no effective methods to control HIR-induced ALI. Desflurane, a widely used inhaled anesthetic, has garnered attention for its protective effects beyond its primary role in maintaining anesthesia (Mangus et al., 2018; Kawanishi et al., 2022); however, the specific molecular mechanisms regulating the effects of desflurane on HIR-induced ALI in young rats have not been fully elucidated. Exploring these underlying mechanisms and identifying safe and effective pharmacological interventions would significantly improve the prognosis of children with such complications.

High-mobility group box 1 (HMGB1), a nuclear protein acting as a damage-associated molecular pattern (DAMP), plays a pivotal role in the pathogenesis of HIR and its associated systemic complications (Tsung et al., 2005; Hua et al., 2019). Specifically, HMGB1 is actively or passively released from the nucleus into the cytoplasm and subsequently into the extracellular space, contributing to systemic inflammation during the early stages of ALI (Ueno et al., 2004). Pattern recognition receptors, such as Toll-like receptors (TLRs) and receptors for advanced glycation end products (RAGE), are activated by circulating HMGB1 (Weber et al., 2015), initiating downstream signaling cascades that amplify inflammatory responses. Silent information regulator 2-related enzyme 1 (SIRT1), a nicotinamide adenine dinucleotide (NAD)^+^-dependent deacetylase, modulates various physiological processes by regulating the acetylation of histones and non-histone proteins (Zhou et al., 2022). Emerging evidence indicates that SIRT1 can attenuate inflammatory responses by deacetylating and sequestering downstream mediators, such as HMGB1, ultimately mitigating organ damage induced by ischemia-reperfusion injury and sepsis (Gao et al., 2015; Rabadi et al., 2015; Han et al., 2017). These insights highlight the potential of the SIRT1/HMGB1 axis as a viable therapeutic target for managing HIR-induced complications.

Studies have demonstrated that desflurane attenuates neutrophil-mediated inflammation by antagonizing the CXCR2 signaling pathway, thereby protecting against ischemia-reperfusion injury (Müller-Edenborn et al., 2015). Additionally, desflurane has been shown to modulate the Nrf2-Keap1-ARE signaling pathway, reducing oxidative stress and inflammation in renal ischemia-reperfusion injury models (Zheng et al., 2020). Furthermore, desflurane has been reported to improve microcirculation (Cho et al., 2017) and lung function by increasing the PaO_2_/FiO_2_ ratio, reducing the number of macrophages and neutrophils in bronchoalveolar lavage fluid (BALF), and lowering levels of proinflammatory cytokines during the development of ALI/ARDS (Schilling et al., 2007; Lin et al., 2018). These protective effects suggest that desflurane may be beneficial in the therapeutic management of HIR-induced lung injury.

Our previous clinical research established a correlation between elevated serum HMGB1 levels and the development of ALI/ARDS in pediatric patients undergoing living donor liver transplantation (Cao et al., 2023). However, the mechanisms by which desflurane modulates HMGB1 nuclear-cytoplasmic translocation and its upstream regulatory pathways, particularly in HIR-induced lung injury, remain poorly defined. Given that pediatric lungs are more susceptible to ischemic injury than adult lungs, our study employed a 70% hepatic warm ischemia-reperfusion model in 2 week-old Sprague-Dawley (SD) rats and NR8383 cell model of oxygen and glucose deprivation (OGD) to simulate the HIR process in pediatric liver transplantation. The primary objective of this study was to evaluate the protective efficacy of desflurane against HIR-induced ALI and explore its underlying regulatory mechanisms. Specifically, this study investigated the relationship between desflurane and HMGB1 nuclear-cytoplasmic translocation, with particular emphasis on the deacetylase activity of SIRT1. The functional necessity of SIRT1 was further validated using its specific inhibitor, Ex527. Collectively, this study provides novel mechanistic insights into the pathophysiology of desflurane and offers potential therapeutic strategies for the prevention and management of ALI/ARDS in pediatric patients undergoing liver transplantation, ultimately optimizing clinical outcomes and long-term quality of life.

## 2 Materials and methods

### 2.1 Reagents

Desflurane (DES, Cat. No. 1001964134) was purchased from Baxter Healthcare Corporation, United States. Ex527 (Cat. No. HY-15452) was supplied by MedChemExpress (Monmouth Junction, NJ, United States). ELISA kits for HMGB1 (Cat. No. TAE-335R), MIP-2 (Cat. No. TAE-497R), and TNF-α (Cat. No. TAE-569R) were obtained from Anoric Biotechnology (Tianjin, China). The albumin detection kit (Cat. No. TC0563) was from Leagene (Beijing, China). Nuclear and cytoplasmic protein extraction kits (Cat. No. WLA020), Cell Counting Kit-8 (CCK-8, Cat. No. WLA074), polyclonal rabbit anti-β-actin (Cat. No. WL01372), and monoclonal rabbit anti-Lamin B1 (Cat. No. WL01775) were supplied by Wanlei Biotechnology (Shenyang, China). Monoclonal rabbit anti-HMGB1 (ab79823) and anti-SIRT1 (ab110304) antibodies were procured from Abcam (Cambridge, UK). Molyclonal mouse anti-TLR4 (Cat. No. 14358), molyclonal rabbit anti-NF-κB p65 (Cat. No. 8242), anti-acetylated-lysine (Cat. No. 9441), anti-MyD88 (Cat. No. 4283), and anti-RAGE (Cat. No. 6996) antibodies were obtained from Cell Signaling Technology (Danvers, MA, United States). Polyclonal rabbit anti-Ly-6G (Cat. No. A22270), anti-Acetyl-HMGB1-K29 (Cat. No. A16002), and anti-Acetyl-HMGB1-K177 (Cat. No. A16005) antibodies were sourced from ABclonal (Wuhan, China). Goat anti-rabbit IgG conjugated with Cy3 (Cat. No. GB21303) and goat anti-mouse IgG conjugated with FITC (Cat. No. GB22301) were obtained from Servicebio (Wuhan, China).

### 2.2 Animals

Male Sprague Dawley (SD) rats (2 weeks-old, weight 25–30 g) were kept under specific-pathogen-free conditions at controlled temperature and humidity and on a 12 h light/12 h dark cycle with *ad libitum* access to water and a standard laboratory diet. Rats were purchased from Beijing Huafukang Bioscience Co., Ltd. (Beijing, China). All animal procedures were approved by the Ethics Committee of Nankai University (approval number: 2020N198KY).

### 2.3 HIR-induced acute lung injury model establishment and drug administration

A rat model of 70% warm HIR was established based on a previously described protocol with minor modifications (Liu et al., 2019). Briefly, the rats were weighed and anesthetized with esketamine (90 mg/kg) and diazepam (5 mg/kg) intraperitoneally. Following midline laparotomy, partial (70%) hepatic warm ischemia was induced by atraumatic vascular occlusion of the portal triad (comprising the hepatic artery, portal vein, and bile duct) supplying the median and left lateral lobes for 90 min. Sham group animals only underwent an identical surgical procedure and dissection of the portal triad without vascular clamping. Throughout the surgical intervention, normothermia was meticulously maintained at 37.0 °C ± 0.5 °C using a feedback-controlled heating blanket.

For the desflurane treatment groups, animals were administered L-DES (5.4%–5.7%), M-DES (9.1%–9.5%), or H-DES (14.5%–15.0%) for 45 min prior to the onset of liver ischemia respectively to explore the dose response; L-DES (5.4%–5.7%) was selected for subsequent experiments. Additionally, rats in the Ex527 group received an intraperitoneal injection of Ex527 (50 mg/kg) 2 h before the modeling. The remaining groups were administered an equivalent volume of vehicle as a control.

### 2.4 Cell culture and oxygen glucose deprivation (OGD) model construction

NR8383 rat alveolar macrophage cell line was purchased from the Cell Bank of the Chinese Academy of Sciences (Shanghai, China). Cells were cultured in Ham’s F-12K medium (Gibco, Invitrogen, United States) supplemented with 15% heat-inactivated fetal bovine serum (Gibco, Invitrogen, United States) and 1.0 mmol/L sodium pyruvate. The cultures were incubated at 37 °C in a humidified atmosphere containing 5% CO_2_. To establish an *in vitro* oxygen-glucose deprivation (OGD) reoxygenation model, the complete growth medium was replaced with glucose-free Ham’s F-12K medium, and the cells were transferred to a hypoxic incubator (1% oxygen, 94% N_2_, and 5% CO_2_) at 37 °C. Following a predetermined duration of hypoxia (optimized via the CCK-8 assay), the cells were re-exposed to the original culture medium and normoxic conditions for 24 h to simulate reoxygenation. Cells were treated with specified concentrations of Ex527 and desflurane (optimal concentrations predetermined using the CCK-8 assay).

As described in the *in vivo* experimental design, desflurane (at a concentration equivalent to L-DES) and/or Ex527 (final concentration of 100 μM) was administered prior to OGD exposure to evaluate their protective effects. DMSO (final concentration ≤0.1% v/v) served as the vehicle control for Ex527. The detailed experimental timeline and treatment groups are illustrated in Figure 7A.

### 2.5 Cell viability

Cell viability was assessed using the Cell Counting Kit-8 (Wanlei Bio, Shenyang, China) according to the manufacturer’s protocol. NR8383 cells were seeded into 96-well plates at a density of 1 × 10^4^ cells/well and cultured in the specified growth medium at 37 °C in a 5% CO_2_ incubator. After the cells reached the desired confluence, they were exposed to different concentrations of desflurane (or Ex527) for 24 h. Subsequently, 10 μL of CCK8 reagent was added, followed by additional incubation at 37 °C for 2 h in a 5% CO_2_ incubator. The absorbance of each well was measured at 450 nm. Cell viability was calculated as a percentage relative to that of the control group.

### 2.6 Measurement of the levels of AST and ALT in young rats

The AST and ALT levels in the serum of young rats were automatically determined using an automatic biochemistry analyzer (Shenzhen Mindray Bio-Medical Electronics Co., Ltd.).

### 2.7 Measurement of the water ratio in lung tissue

The middle lobe of the lung was resected, blotted dry with filter paper, and its weight was recorded. The lobe was then dried in an 80 °C oven for 48 h. The degree of pulmonary edema was evaluated using the water content ratio, which was calculated as follows: ([wet weight−dry weight]/wet weight) × 100%.

### 2.8 Histological analysis

For histopathological evaluation, formalin-fixed liver or lung tissues were dehydrated and embedded in paraffin. The paraffin blocks were cut into 5-μm sections and subjected to standard hematoxylin and eosin (H&E) staining. Suzuki’s scores and lung injury scores were determined by a blinded pathologist using ten random fields (×200). Liver injury was based on scores of 0 (no injury), 1 (little), 2 (mild), 3 (moderate), or 4 (severe) in the presence of hepatocellular congestion and necrosis with pyknosis. Similarly, lung injury was based on scores of 0 (no injury), 1 (< 25% involved), 2 (25%–50% involved), 3 (50%–75% involved), or 4 (> 75% involved) in the presence of alveolar fibrin/edema, alveolar hemorrhage, septal thickening, and cellular infiltration. Suzuki’s scores and lung injury scores were determined in accordance with previously established criteria (Suzuki et al., 1993; Zhao et al., 2015).

### 2.9 Immunohistochemistry and immunofluorescence

After being fixed with 4% paraformaldehyde, lung tissues were processed through a graded dehydration series and embedded into paraffin. To visualize specific proteins, lung sections were subjected to immunohistochemistry using antibodies for HMGB1 (1:350) and SIRT1 (1:250). For semi-quantitative analysis, digital images were evaluated using ImageJ software (National Institutes of Health, Bethesda, MD, United States). The Mean Optical Density (MOD) was used to represent the relative expression levels of the target proteins. Specifically, MOD was calculated as the ratio of the Integrated Optical Density (IOD) of the positive staining region to the total measured positive area (MOD = IOD/ Area). For cellular immunofluorescence, permeabilization was achieved with 0.1% Triton X-100, followed by blocking in 10% BSA. Primary antibody incubation (Ly6G, 1:150; HMGB1, 1:250; and TLR4, 1:250) was conducted at 4 °C for 12 h. Subsequent to PBST rinses, secondary detection was performed using either Cy3-conjugated goat anti-rabbit (1:300) or FITC-conjugated goat anti-mouse IgG (1:200) for 1 h at ambient temperature in the dark. Finally, DAPI (Sigma-Aldrich, St. Louis, United States) was applied for nuclear visualization, and the staining patterns were captured using a fluorescence imaging system (Olympus, Tokyo, Japan). To verify the specificity of immunofluorescence signals and rule out interference caused by non-specific binding and tissue autofluorescence, appropriate negative controls were set up. Primary antibodies were omitted in these controls, and no detectable non-specific fluorescent signals were observed under identical imaging acquisition settings (Supplementary Figure S1).

### 2.10 Collection of bronchoalveolar lavage fluid and ELISAs

Young rats were euthanized by cervical dislocation and sterilized with 75% ethanol for approximately 10 min. Following a midline neck incision to expose the trachea, a truncated indwelling needle was inserted for tracheal intubation. Bronchoalveolar lavage was performed by instilling ice-cold sterile saline followed by repeated gentle aspiration. The recovered BALF was centrifuged at 500 rpm for 5 min at 4 °C. The supernatant was collected and stored at 4 °C for albumin detection (Leagene, Beijing, China) or at −80 °C for subsequent cytokine quantification. The levels of TNF-α (Absin Bioscience, China) and MIP-2 (MultiSciences, Hangzhou, China) were determined using ELISA kits, according to the manufacturer’s instructions.

### 2.11 Nucleus and cytoplasm protein isolation

Nuclear and cytoplasmic protein fractions were isolated from lung tissues and cells using a Nuclear and Cytoplasmic Protein Extraction Kit (Wanlei Bio, Shenyang, China) in strict accordance with the manufacturer’s protocol. Briefly, samples were rinsed twice and harvested in ice-cold phosphate-buffered saline (PBS). After a preliminary centrifugation at 2000 g for 5 min, the resulting pellet was lysed in 1 mL of extraction reagent for 5 min. The lysates were subsequently clarified by centrifugation at 14,000 × *g* for 10 min at 4 °C, after which the supernatants containing the target proteins were recovered for downstream applications.

### 2.12 Western blotting

Total protein from lung tissues and NR8383 cells was extracted using RIPA lysis buffer supplemented with a protease and phosphatase-inhibitor cocktail. Protein concentrations were quantified using a BCA Protein Assay Kit (Pierce, Thermo Fisher Scientific, United States). Equal amounts of protein were separated by sodium dodecyl sulfate–polyacrylamide gel electrophoresis and transferred to polyvinylidene difluoride membranes. The membranes were blocked with 5% dry non-fat milk for 1 h at room temperature and then incubated overnight at 4 °C with primary antibodies against the following targets: HMGB1 (1:10,000) and SIRT1(1:6,000), TLR4 (1:5,000), RAGE (1:1,000), MyD88 (1:600), NF-κB p65 (1:1,000), Acetylated-lysine (1:1,000), anti-Acetyl-HMGB1-K29 (1:1,000), anti-Acetyl-HMBG1-K177 (1:1,000), Lamin B (1:600), or β-actin (1:1,000). The next day, the membranes were washed and incubated with corresponding horseradish peroxidase-coupled secondary antibodies (1:2000). Protein bands were visualized using an enhanced chemiluminescence (ECL) detection system (Merck, Darmstadt, Germany), and densitometric analysis was performed using the Bio-Rad ChemiDoc imaging system (Hercules, CA, United States). β-actin and Lamin B served as loading controls for total/cytoplasmic and nuclear fractions, respectively. Relative protein expression levels were normalized to those of the control group.

### 2.13 Co-immunoprecipitation and protein acetylation determination

The acetylation levels of HMGB1 in young rat lung tissues and NR8383 cells were determined using an immunoprecipitation (co-immunoprecipitation) kit (Elabscience, Wuhan, China), according to the manufacturer’s instructions. Briefly, total protein was extracted from the lung tissues/cells as previously described. Protein A/G affinity agarose beads were pre-washed with 1× PBS and incubated with an anti-HMGB1 primary antibody (1:100) at room temperature for 10 min to facilitate the formation of antibody–antigen complexes in solution. Protein A/G agarose beads were added and incubated for 2 h at 4 °C with gentle agitation to capture the complexes. After five washes to remove non-specific proteins, the bead-bound proteins were eluted using a denaturing method by adding 1× SDS loading buffer and boiling at 95 °C for 5 min. Finally, the samples were analyzed by western blotting using an anti-acetyl-lysine antibody (1:100) to evaluate the acetylation levels of HMGB1.

### 2.14 RNA isolation and detection of mRNAs

The TRIzol reagent (Takara Bio, Shiga, Japan) was used to isolate total RNA from rat lung and cells following the manufacturer’s instructions. cDNA was synthesized using the PrimeScript RT Reagent Kit (Tiangen Biotech, Beijing, China) for mRNA quantification and a microRNA Reverse Transcription Kit (Tiangen Biotech, Beijing, China) for miRNA. Real-time PCR was implemented in a 10-μL reaction mixture comprising cDNA, specific primers, and SYBR Green Master Mix (Tiangen Biotech, Beijing, China). For every biological sample, four technical replicates were performed to verify experimental consistency. Normalization of mRNA and miRNA expression was conducted relative to the endogenous controls U6 small nuclear RNA and β-actin, respectively. Relative gene expression was calculated using the 2^−ΔΔCT^ method. The specific primer sequences are detailed in Table 1.

**TABLE 1:** RNA primers.

Primer name	Sequence (5’ to 3’)
SIRT1	​
Forward primer	TGA​TCC​GAG​ATG​TGG​AAC​TGG
Reverse primer	CTC​CTC​CGC​TTG​GTG​GTT​T
HMGB1	​
Forward primer	GGA​GTG​GCT​TTT​GTC​CCT​CAT
Reverse primer	TGC​CTC​TCG​GCT​TTT​TAG​GA
TNF-α	​
Forward primer	TGA​TCC​GAG​ATG​TGG​AAC​TGG
Reverse primer	CTC​CTC​CGC​TTG​GTG​GTT​T
IL-1β	​
Forward primer	CAC​TGT​GGC​TGT​GGT​CAC​CTA​TC
Reverse primer	ACT​GAC​ACT​CCG​CAC​AAA​GCA​G
β-actin	​
Forward primer	CGT​TGA​CAT​CCG​TAA​AGA​CC
Reverse primer	AAC​AGT​CCG​CCT​AGA​AGC​AC

### 2.15 Statistical analysis

Statistical analyses and data visualizations were performed using GraphPad Prism software (version 9.0; GraphPad Software, Inc., San Diego, CA, United States). Continuous variables with a normal distribution are expressed as the mean ± standard error of the mean (SEM). Multiple group comparisons were performed using one-way analysis of variance (ANOVA), followed by Tukey’s multiple comparison test. Categorical data were presented as percentages and analyzed using the chi-square test or Fisher’s exact test. *P* < 0.05 was considered statistically significant.

## 3 Results

### 3.1 HIR-induced ALI peaked at 6 h in young rats

**FIGURE 1:**
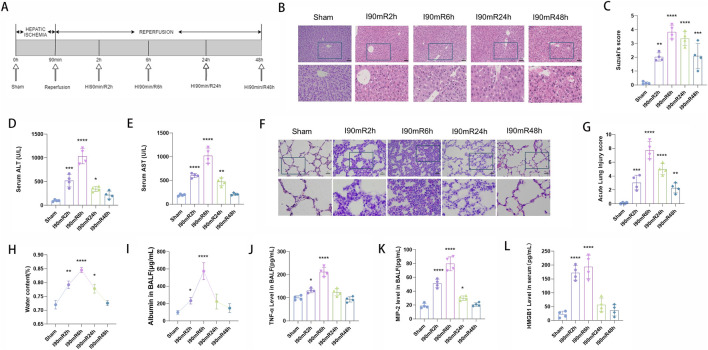
Successful establishment of HIR-induced acute lung injury model in young rats. **(A)** Schematic of the animal model. **(B)** Representative images of hematoxylin and eosin (HE)-stained liver tissue in different groups (×400 magnification). Scale bar, 50 μm. **(C)** Suzuki’s pathological score was evaluated. **(D,E)** Hepatocellular function in serum samples was evaluated by detecting ALT and AST levels. **(F)** Representative images of H&E-stained lung tissue in different groups (×400 magnification). Scale bar, 50 μm. **(G)** Acute lung injury score. **(H)** Lung water content. **(I)** Albumin in BALF. **(J)** TNF-α in BALF. **(K)** MIP-2 in the BALF. **(L)** HMGB1 in serum. One-way analysis of variance with Tukey’s multiple comparison test was used for the analysis. Graphs represent means ± SEM, n ≥ 4; *P < 0.05, **P < 0.01, ***P < 0.001, ****P < 0.0001, compared with the sham group.

To determine the effects of HIR on lung injury in young rats, pulmonary and hepatic tissues were harvested at different time points after the successful establishment of the HIR model. A schematic diagram of the HIR model in young rats is shown in Figure 1A. As shown in Figures 1B–E, liver injury reached its peak at 6 h after HIR, as evidenced by maximal Suzuki’ scores and the highest serum levels of ALT and AST. Concurrently, lung injury also peaked at this time point, as indicated by the highest acute lung injury scores, peak lung water content, and maximal levels of albumin, TNF-α, and MIP-2 in the BALF (Figures 1F–K). Furthermore, systemic HMGB1 levels peaked at the 6 h after HIR as shown in Figure 1L. Considering the confluence of pathological damage, pulmonary edema, and proinflammatory mediators, we postulated that the 6 h time point represented the period of the most severe lung injury after HIR. Accordingly, all subsequent experiments were conducted 6 h after the reperfusion.

### 3.2 L-DES and M-DES could attenuate HIR-induced ALI in young rats

**FIGURE 2:**
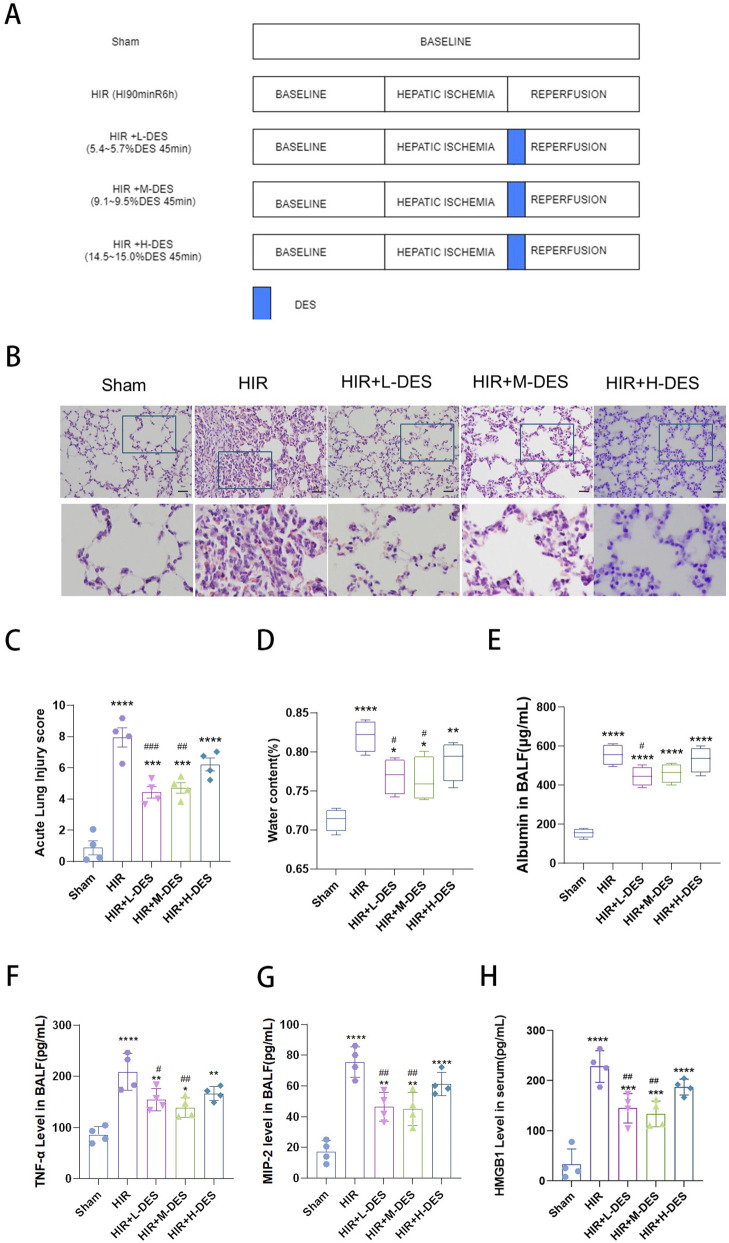
Desflurane treatment restores HIR-induced acute lung injury in young rats. **(A)** Different concentrations of desflurane treatment in each group of young rats. **(B)** Representative images of H&E-stained lung tissue in different groups (×400 magnification). Scale bar, 50 μm. **(C)** Acute lung injury score. **(D)** Lung water content. **(E)** Albumin in BALF. **(F)** TNF-α levels in BALF. **(G)** MIP-2 in BALF. **(H)** HMGB1in serum. One-way analysis of variance with Tukey’s multiple comparison test was used for the analysis. Graphs represent means ± SEM, n ≥ 4; *P < 0.05, **P < 0.01, ***P < 0.001, ****P < 0.0001, compared with the sham group; ^#^ *P* < 0.05, ^##^ *P* < 0.01, ^###^ *P* < 0.001 compared with the HIR group.

Next, to evaluate the effects of desflurane on young rats, the animals were assigned to L-DES, M-DES and H-DES groups according to the concentration of desflurane administered. A schematic diagram of desflurane treatment in young rats is shown in Figure 2A. As shown in Figures 2B,C, both the L-DES and M-DES groups alleviated lung tissue pathological damage and acute lung injury scores after HIR. Both groups also showed decreased lung water content and lower concentrations of albumin, TNF-α, and MIP-2 in BALF (Figures 2D–G). Furthermore, serum HMGB1 protein levels were significantly reduced in both the L-DES and M-DES groups after HIR as shown in Figure 2H. Based on economic and efficiency considerations, L-DES group was selected for subsequent experiments.

### 3.3 Desflurane inhibits nucleoplasmic translocation in lung tissue of young rats after HIR

**FIGURE 3:**
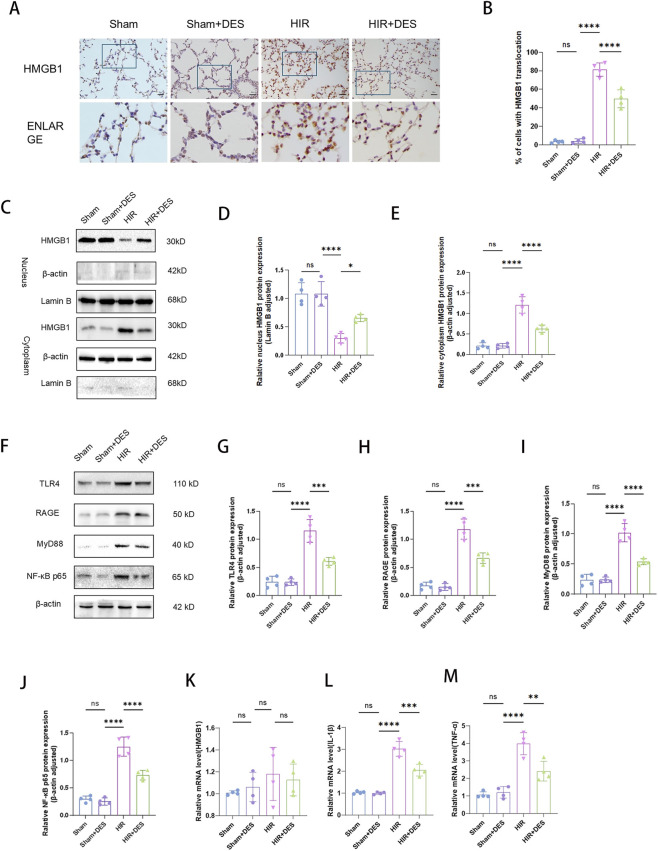
Desflurane inhibits HMGB1 cytoplasmic location and its downstream signaling pathway in lung tissue after HIR. **(A)** Representative immunohistochemical staining of HMGB1 in lung sections (magnification, ×200). Scale bar, 100 μm. **(B)** Rate of cells with HMGB1 translocation. **(C–E)** Representative western blots and quantification of nuclear and cytoplasmic HMGB1 in lung tissues. **(F–J)** Representative western blots and quantification of TLR4, RAGE, MyD88, and NF-κB p65 in lung tissues. **(K–M)** Measurement of proinflammatory cytokine mRNAs (HMGB1,IL-1β, and TNF-α) levels in lung tissues by RT-qPCR. One-way analysis of variance with Tukey’s multiple comparison test was used for the analysis. Graphs represent means ± SEM, n ≥ 4; *P < 0.05, **P < 0.01, ***P < 0.001, ****P < 0.0001.

**FIGURE 4:**
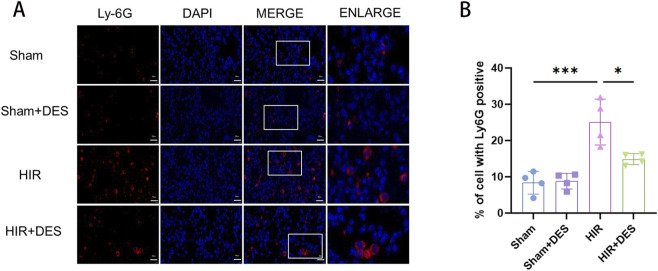
Desflurane reduced the rate of Ly-6G^+^ cells in lung tissue after HIR. **(A)** Representative immunofluorescence images of Ly-6G (red) and DNA staining (blue) in lung sections (×400 magnification). Scale bar, 20 μm. **(B)** Rate of Ly-6G^+^ cells. One-way analysis of variance with Tukey’s multiple comparisons test was used for analysis. Graphs represent means ± SEM, n ≥ 4; *P < 0.05, ***P < 0.001.

Having established the protective effect of desflurane against HIR-induced ALI, we next sought to investigate the molecular mediators driving this effect. These results showed an increase in circulating HMGB1 levels in juvenile rats after HIR. Therefore, we proceeded to investigate HMGB1 expression within the lung tissue. As shown in Figures 3A,B, HMGB1 levels were increased in the lung tissue of young rats after HIR. Desflurane treatment significantly increased nuclear HMGB1 expression while decreasing its cytoplasmic levels after HIR (Figures 3C–E). Additionally, the protein levels of TLR4, RAGE, MyD88, and NF-κB p65 were similar between the sham and sham + DES groups, increased markedly in the HIR group, and partially reversed by desflurane in the HIR + DES group (Figures 3F–J). As shown in Figures 3K–M, HMGB1 mRNA levels did not change significantly after HIR, whereas IL-1β and TNF-α mRNA levels increased significantly; desflurane treatment significantly reduced IL-1β and TNF-α mRNA levels. Figures 4A,B demonstrate that the percentage of Ly-6G^+^ cells in lung tissue increased significantly after HIR and significantly decreased after desflurane treatment. These results indicate that desflurane could attenuate the HMGB1 expression and downstream signals in the juvenile lung after HIR.

### 3.4 Desflurane upregulated the levels of SIRT1 and the acetylation of HMGB1 in the lung tissue of young rats after HIR

**FIGURE 5:**
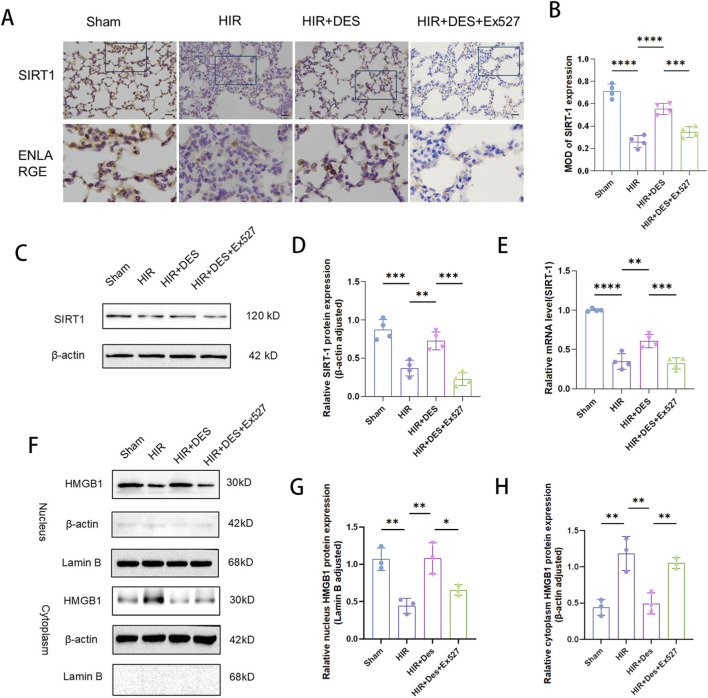
Desflurane upregulates SIRT1 signaling and translocation of HMGB1 in lung tissue of young rats after HIR. **(A)** Representative immunohistochemical staining of SIRT1 in lung sections (×400 magnification). Scale bar, 50 μm. **(B)** Semi-quantitative analysis of SIRT1 expression, presented as Mean Optical Density (MOD). **(C,D)** Representative western blots and quantification of SIRT-1 in lung tissues. **(E)** Measurement of SIRT-1 mRNAs in lung tissues by RT-qPCR. **(F–H)** Representative western blots and quantification of nuclear and cytoplasmic HMGB1 in lung tissues. One-way analysis of variance with Tukey’s multiple comparisons test was used for analysis. Graphs represent means ± SEM, n ≥ 3; **P < 0.01, ***P < 0.001, ****P < 0.0001.

**FIGURE 6:**
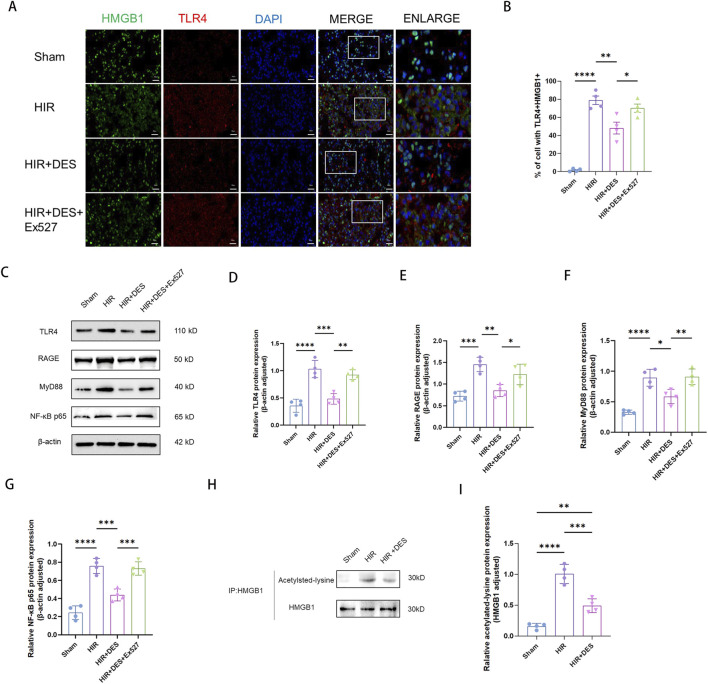
Desflurane reverses the translocation and acetylation of HMGB1 in lung tissue of young rats after HIR. **(A,B)** Representative immunofluorescence images of HMGB1 (green) and TLR4 (red), and DNA staining (blue) in lung sections (×400 magnification). Scale bar, 20 μm. **(C–G)** Representative western blots and quantification of TLR4, RAGE, MyD88, and NF-κB p65 in lung tissues. **(H,I)** IP: Representative western blots and quantification of acetylsied-HMGB1 in lung tissues. One-way analysis of variance with Tukey’s multiple comparisons test was used for analysis. Graphs represent means ± SEM, n ≥ 4; *P < 0.05, **P < 0.01, ***P < 0.001, ****P < 0.0001.

Given the pivotal role of SIRT1 in orchestrating the inflammatory response, we examined whether desflurane maintains pulmonary homeostasis by modulating the SIRT1/HMGB1 axis. To determine whether SIRT1 mediates the effects of desflurane on HMGB1 nucleocytoplasmic translocation, a selective SIRT1 inhibitor, Ex527, was employed. As shown in Figures 5A–E, immunohistochemical staining, western blots, and RT-qPCR showed that HIR significantly downregulated SIRT1 protein expression, whereas desflurane treatment partially restored SIRT1 levels; this upregulation was reversed by the selective SIRT1 inhibitor Ex527. Figures 5F–H reveals that Ex527 reversed the desflurane-induced retention of HMGB1 in the nucleus, as evidenced by a significant decrease in nuclear HMGB1 and an increase in cytoplasmic HMGB1 levels. As shown in Figures 6A,B, HIR markedly upregulated TLR4 expression and facilitated HMGB1 nuclear-to-cytoplasmic translocation. Desflurane partially reversed these alterations, whereas co-administration of the selective SIRT1 inhibitor Ex527 reversed the protective effects of desflurane. The percentage of TLR4^+^HMGB1^+^ cells mirrored these findings. Consistent trends were observed in the expression of downstream signaling pathways, including TLR4, RAGE, MyD88, and NF-κB p65 (Figures 6C–G). Furthermore, desflurane significantly reduced the acetylation levels of HMGB1 in lung tissue after HIR (Figures 6H,I). These findings suggest that the restoration of SIRT1 expression is a component of the pharmacological action of desflurane in the juvenile lung.”

### 3.5 Desflurane attenuates inflammation in NR8383 cells after OGD *in vitro*.

**FIGURE 7:**
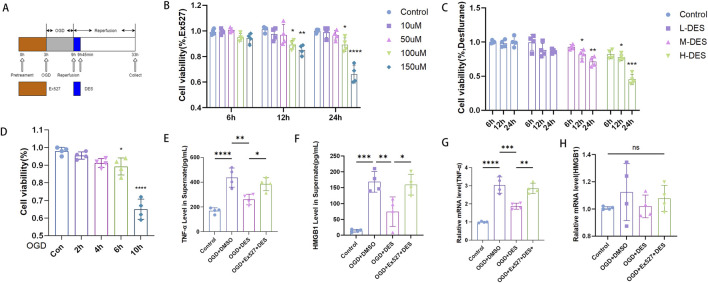
Desflurane attenuates inflammation in NR8383 cells *in vitro*. **(A)** Flowchart of the OGD treatment model *in vitro*. **(B)** Cell viability of NR8383 cells was detected with different concentrations of Ex527 by CCK-8. **(C)** Cell viability of NR8383 cells was detected with different concentrations of desflurane by CCK-8. **(D)** Cell viability of NR8383 cells was assessed of OGD treatment duration by the CCK-8 assay. **(E)** TNF-α levels in the supernatant. **(F)** HMGB1 levels in the supernatant. **(G)** Measurement of TNF-α mRNAs levels in NR8383 cells by RT-qPCR. **(H)** Measurement of HMGB1 mRNAs levels in NR8383 cells by RT-qPCR. One-way analysis of variance with Tukey’s multiple comparisons test was used for analysis. Graphs represent means ± SEM, n ≥ 4; *P < 0.05, **P < 0.01, ***P < 0.001, ****P < 0.0001.

Concurrently, an OGD model of NR8383 cells was established *in vitro* to verify the inflammatory response to desflurane. The experimental flowchart of the OGD protocol was shown in Figure 7A. CCK-8 assays were performed to determine the optimal OGD duration and appropriate concentrations of Ex527 and desflurane (Figures 7B–D). Cell viability was significantly reduced after 24 h of treatment with 100 μM Ex527. A similar significant decline in cell viability was observed after 24 h of exposure to L-DES. Cell viability was preserved at 6 h after OGD but declined significantly at 10 h after OGD. Based on these findings, the corresponding drug concentrations and time points were used in all subsequent experiments. Additionally, as shown in Figures 7E–H, TNF-α and HMGB1 levels in the supernatant were significantly increased after OGD, attenuated by desflurane treatment, and subsequently reversed by Ex527. Corresponding changes were also observed at the mRNA levels except HMGB1. These results suggest that desflurane reduces the inflammatory response elicited by OGD in NR8383 cells.

### 3.6 Desflurane inhibits HMGB1 nucleoplasmic translocation and expression of downstream signaling pathways by upregulating SIRT1 in NR8383 cells

**FIGURE 8:**
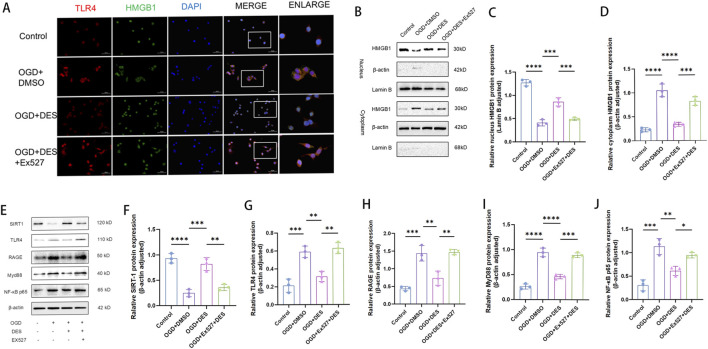
Desflurane inhibits HMGB1 nucleoplasmic translocation and signaling pathways in NR8383 cells. **(A)** Representative immunofluorescence of HMGB1 (green) and TLR4 (red), and DNA staining (blue) in NR8383 cells. Scale bar, 20 μm. **(B–D)** Representative western blots and quantification of nuclear and cytoplasmic HMGB1 in NR8383 cells. **(E–J)** Representative western blots and quantification of TLR4, RAGE, MyD88, and NF-κB p65 in NR8383 cells. One-way analysis of variance with Tukey’s multiple comparisons test was used for analysis. Graphs represent means ± SEM, n ≥ 3; *P < 0.05, **P < 0.01, ***P < 0.001, ****P < 0.0001.

Subsequently, the effect of desflurane on HMGB1 nucleocytoplasmic translocation and its downstream signaling pathways were examined in NR8383 cells *in vitro*. As shown in Figure 8A, immunofluorescence revealed that HMGB1 underwent significant nucleocytoplasmic translocation in NR8383 cells after OGD. Desflurane treatment increased nuclear HMGB1 levels, as evidenced by increased nuclear expression and a concomitant decrease in cytoplasmic levels (Figures 8B–D). Additionally, western blot analysis (Figures 8E–J) demonstrated that SIRT1 levels were downregulated by OGD reoxygenation but significantly restored by desflurane treatment; this effect was subsequently abolished by Ex527. In contrast, the expression levels of TLR4, RAGE, MyD88, and NF-κB p65 displayed an opposite trend exhibited an inverse pattern, where desflurane-mediated downregulation was reversed upon SIRT1 inhibition. These results are consistent with the findings obtained from the *in vivo*.

### 3.7 Desflurane inhibits HMGB1 acetylation in NR8383 cells via SIRT1

**FIGURE 9:**
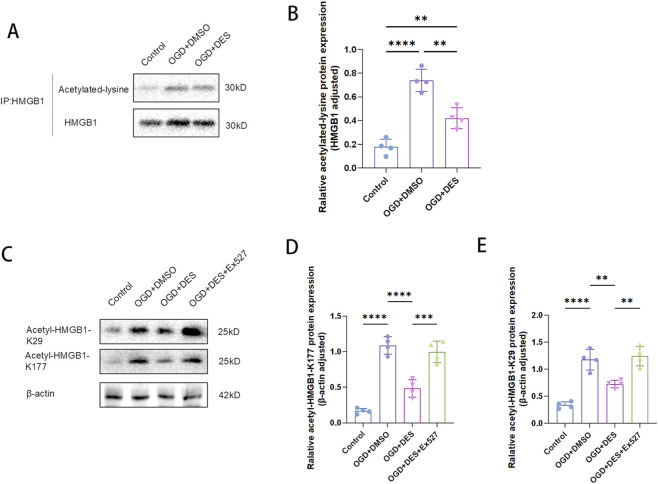
Desflurane inhibits HMGB1 acetylation in NR8383 cells via SIRT1. **(A,B)** IP: Representative western blots and quantification of acetylated HMGB1 in NR8383 cells. **(C–E)** Representative western blots and quantification of Acetyl-HMGB1-K29, K-177 in NR8383 cells. One-way analysis of variance with Tukey’s multiple comparison test was used for the analysis. Graphs represent means ± SEM, n ≥ 4; **P < 0.01, ***P < 0.001, ****P < 0.0001.

Finally, we assessed the effect of desflurane on HMGB1 acetylation in NR8383 cells. As shown in Figures 9A,B, total HMGB1 acetylation was increased significantly after OGD reoxygenation and was markedly attenuated by desflurane treatment. Furthermore, the acetylation sites K29 and K177 of HMGB1 were also detected, with the results shown in Figures 9C–E. Acetyl-HMGB1-K29 and Acetyl-HMGB1-K177 levels increased significantly after OGD reoxygenation. These increases were suppressed by desflurane, but were subsequently restored following Ex527 treatment. These findings provide further insight into the underlying mechanisms.

## 4 Discussion

ALI/ARDS is a prevalent respiratory complication after pediatric liver transplantation and significantly affects the postoperative survival of pediatric patients. Although its underlying mechanisms are complex and heterogeneous, accumulating evidence shows that excessive inflammatory responses are critical mediators of ALI/ARDS progression. In this study, we observed that desflurane post-conditioning significantly alleviated HIR-induced ALI in young rats. This protective effect is mediated by the upregulation of SIRT1 and subsequent inhibition of HMGB1 acetylation and release.

Although the protective effects of volatile anesthetics against lung injury have been extensively reported in studies using adult animal models (O’Gara and Talmor, 2016), there is a paucity of data on animal models at the juvenile stage. Compared to adults, infants undergoing liver transplantation are more susceptible to ALI (Sucre et al., 2020), which can result in persistent physiological deficits. Nevertheless, recent studies have shown that the mechanisms of pulmonary damage and regeneration occur in an age-dependent manner (Penkala et al., 2021). The pulmonary vascular bed is the primary target of proinflammatory mediators from the liver graft. The damaged liver releases a cascade of cytokines and proinflammatory mediators after reperfusion, thereby activating pulmonary innate immunity. In our study, 2 week-old SD rats were used to establish a model of 70% warm HIR to evaluate the protective effect of desflurane on lung injury. Both hepatic and pulmonary injury peaked in severity at 6 h after reperfusion, which is consistent with the results of previous studies (Lepagnot, 2015; Yao et al., 2017).

Recent evidence has demonstrated that both sevoflurane preconditioning and post-conditioning significantly improve oxygenation, reduce pulmonary edema, and decrease IL-1β, IL-6, and TNF-α levels in lung transplant recipients, with no marked differences in efficacy between the two strategies (Ohsumi et al., 2017). Another study indicated that only post-conditioning can attenuate the systemic inflammatory response in HIR models (Figueira et al., 2019). Based on this evidence, we administered desflurane immediately after reperfusion for 45 min. Our results showed that both L-DES and M-DES effectively mitigated acute lung injury following HIR. Notably, Strosing et al. (2016) reported that desflurane failed to exert a protective effect against ventilator-induced lung injury in mice that is seen with isoflurane and sevoflurane. However, only a single concentration of desflurane was used in this study, which may have limited the assessment of its therapeutic potential. Specifically, one study reported that desflurane can reduce neutrophil accumulation by inhibiting the CXCR2 signaling pathway, thereby exerting a beneficial effect during ischemia-reperfusion (Müller-Edenborn et al., 2015). The present study also supports this finding: low and medium concentrations of desflurane decreased the level of MIP-2 in the BALF of young rats. MIP-2, also known as CXCL2, is a specific chemokine for CXCR2. It recruits neutrophils to the injury site during the inflammatory response and mediates inflammatory effects.

Another study demonstrated that desflurane significantly alleviates lung injury in mice with endotoxemia by inhibiting the IκB-α degradation pathway in alveolar macrophages, which in turn inhibits inflammatory signaling pathways and exerts anti-inflammatory effects (Boost et al., 2006). Additionally, clinical studies have confirmed that desflurane can reduce the concentration of proinflammatory mediators in BALF (Schilling et al., 2011), which is consistent with our findings.

Although desflurane attenuates lung injury, pulmonary edema, and endothelial permeability by downregulating proinflammatory cytokines, we unexpectedly observed that this protective effect was not strictly dose-dependent: H-DES failed to confer additional benefits over M-DES. The loss of efficacy at higher concentrations is likely driven by a dual mechanism involving systemic and local adverse effects. Systemically, high-dose desflurane is known to induce sympathetic hyperactivity, leading to catecholamine surges that promote endothelial dysfunction, increased vascular permeability, and pro-inflammatory signaling (Hoeppner, 2022). Locally, excessive concentrations of volatile anesthetics may exert direct alveolar cytotoxicity by disrupting lipid bilayers, impairing mitochondrial respiratory function, and triggering a Ca^2+^-driven oxidative burst (Allaouchiche et al., 2001). These combined systemic and local stressors likely overwhelm the protective capacity of the SIRT1/HMGB1 signaling axis for desflurane post-conditioning in juvenile models. In adult ICU practice, the optimal MAC of desflurane for sedation is approximately 0.6 (Wetterkamp et al., 2021). A similar bell-shaped dose–response curve has been described for the lung-protective effects of isoflurane (Fujinaga et al., 2006). Collectively, desflurane (and potentially other volatile anesthetics) requires balancing its beneficial effects against dose-related adverse effects. Careful titration is essential, especially in pediatric patients, where safe concentrations have not yet been definitively established.

HMGB1 is a ubiquitous nuclear protein that predominantly localizes to the nucleus under normal physiological conditions. HIR injury upregulates the expression of HMGB1 receptors, such as TLR4 and RAGE, in ischemic tissues. This process is accompanied by increased expression of proinflammatory cytokines, including TNF-α and IL-6, and promotes the nucleocytoplasmic translocation of HMGB1, along with enhanced local cytokine production (Fujinaga et al., 2006). Our study revealed prominent nucleocytoplasmic translocation of HMGB1 in most pulmonary cells following HIR injury. However, desflurane post-conditioning significantly reduced the percentage of cells exhibiting HMGB1 nuclear-to-cytoplasmic translocation. The receptors of HMGB1 (TLR4 and RAGE) and their downstream signaling components mirrored these changes, suggesting that the translocation of HMGB1 is a key event in activating downstream inflammatory cascades. These findings suggest that circulating HMGB1 induces NF-κB activation and upregulates inflammatory mediators through the TLR4/RAGE/MyD88 signaling axis, which is consistent with previous studies (Prantner et al., 2020). Genetic deletion of TLR4 attenuated pulmonary cytokine release, reversed the histopathological manifestations of lung injury after rat orthotopic liver transplantation, and ultimately improved survival (Chi et al., 2015). Furthermore, our immunofluorescence analysis indicated that desflurane reduced the infiltration of Ly-6G^+^ inflammatory cells in lung tissues. These data suggest that HIR-induced lung injury in juvenile rats is, at least in part, mediated by HMGB1-driven activation of the TLR4/RAGE/MyD88/NF-κB axis, with consequent overproduction of IL-6 and TNF-α.

While the present study focused on the TLR4/ NF-κB axis as the primary downstream signaling pathway of HMGB1, it is essential to acknowledge the broader inflammatory network highlighted by recent multi-omics studies. Substantial evidence indicates that the mitogen-activated protein kinase (MAPK) signaling pathway, alongside cytokines such as IL-17 and TNF, undergoes perturbation during HIR (Liu et al., 2025). Extracellular HMGB1 is an activator of not only NF-κB but also the MAPK cascade via TLR4 and RAGE (Jhun et al., 2015). Although we did not evaluate MAPK activation or IL-17 levels in this study, the prevention of HMGB1 nucleocytoplasmic translocation by desflurane theoretically curtails multiple parallel downstream cascades. Future research should extend these findings by comprehensively profiling the MAPK/IL-17 network to fully map the transcriptomic and proteomic landscape modulated by desflurane-induced SIRT1 activation.

The localization and activity of HMGB1 are influenced by post-translational modifications such as acetylation, phosphorylation, and methylation. Among these modifications, acetylation is closely associated with nucleocytoplasmic translocation (Chen et al., 2022). SIRT1 is an enzyme that mediates deacetylation in an NAD^+^-dependent manner. Recent studies have shown that SIRT1 plays a crucial role in preventing oxidative stress and regulating inflammation, and changes in its expression and activity are associated with inflammatory diseases (Yang et al., 2022). Our results demonstrated that desflurane attenuated the inflammatory response by regulating the degree of HMGB1 nucleocytoplasmic translocation and acetylation via SIRT1, thereby influencing the severity of HIR-induced ALI in young rats. Specifically, desflurane upregulated SIRT1 levels, which led to reduced HMGB1 acetylation levels and increased nuclear accumulation, while simultaneously decreasing cytoplasmic accumulation. These protective effects were reversed by the selective SIRT1 inhibitor EX527. These results indicate that SIRT1 contributes to the release of HMGB1 into the cytoplasm of lung tissues after HIR, and desflurane mediates HMGB1 nucleocytoplasmic translocation and acetylation via SIRT1; this protective effect was reversed following Ex527 treatment. The percentage of HMGB1^+^TLR4^+^ cells and the downstream inflammatory pathways of HMGB1 were also activated, showing similar trends. These findings suggest that desflurane mediates HMGB1 nucleocytoplasmic translocation and acetylation by regulating SIRT1 levels.

Alveolar macrophages (AMs) are central regulators of pulmonary inflammation and the local inflammatory microenvironment, playing a pivotal role in the onset and progression of ALI/ARDS (Woo et al., 2021). These cells can be triggered to release a large array of inflammatory mediators, acting as the igniter of cascading responses and a key driver of cytokine storms. Consequently, targeting AM-derived cytokines is a promising therapeutic strategy. In this study, we employed rat alveolar macrophages (NR8383) to establish an OGD model that recapitulates the principal pathophysiological events encountered by cells during HIR injury *in vitro*. Based on the CCK-8 results, we identified the optimal combination of OGD and recovery duration. Similar to our *in vivo* findings, the selective SIRT1 inhibitor Ex527 confirmed that SIRT1 expression modulates HMGB1 subcellular localization and reduces intracellular acetylation levels, consistent with previous reports (Zhang et al., 2020). Additionally, our research shows that OGD markedly increased the acetylation of HMGB1, whereas desflurane attenuated this interaction. Previous studies have identified lysine residues K29 and K177 as the principal sites targeted by SIRT1-mediated deacetylation of HMGB1 (Wei et al., 2019; Xu et al., 2019). Accordingly, we quantified the acetylation status at these two canonical sites using western blotting. Our results showed that OGD significantly increased acetylation at both residues, which was reversed by desflurane treatment. The present study provides evidence that the protective role of desflurane is driven by SIRT1-dependent deacetylation of HMGB1, with K29 and K177 serving as the targeted acetylation sites.

Our findings have significant clinical implications. Currently, the primary strategy for preventing ALI/ARDS remains intraoperative lung-protective ventilation. Despite substantial advancements in ventilator support and postoperative care, as well as extensive research into the molecular mechanisms of ALI/ARDS, hospitalization and mortality rates in these patients remain persistently high. Consequently, there is an urgent need to develop strategies to preserve pulmonary function and mitigate secondary injuries during HIR. Our study demonstrated that desflurane alleviates HIR-induced ALI in young rats. Importantly, this study may provide insights for future clinical trial designs for the clinical maintenance of volatile anesthetics during pediatric liver transplantation. Specifically, our findings may help inform the optimal timing of volatile anesthetic administration during pediatric liver transplantation. initiating desflurane administration immediately before graft reperfusion could inhibit the acute surge of HMGB1 release. Furthermore, maintaining a low-to-moderate MAC of desflurane during this phase may be sufficient to establish SIRT1/HMGB1-mediated lung protection, maximizing anti-inflammatory benefits while minimizing potential dose-dependent hemodynamic adverse effects in pediatric patients.

This study had several limitations. First, while our pharmacological inhibition protocol demonstrated the involvement of SIRT1, definitive confirmation of functional necessity requires genetic validation. Future investigations should employ SIRT1-overexpressing models to determine if upregulating SIRT1 alone is sufficient to phenocopy the protective effects of desflurane. Regarding downstream targets, since global HMGB1 knockout is often neonatally lethal, conditional HMGB1-deficient models may further clarify the cellular origin and mechanistic contribution of HMGB1-mediated inflammatory signaling. Second, desflurane regulates SIRT1, HMGB1 nucleocytoplasmic translocation, and acetylation, with HMGB1 activating NF-κB to induce inflammation. The link between HMGB1 (a SIRT1 target) and SIRT1-regulated translocation requires further investigation. Third, we focused on lung injury (the most vulnerable remote organ) but did not assess other organ or immune damage post-HIR. Alveolar epithelial/endothelial cells passively release HMGB1 during HIR; however, advanced methods are needed to identify its cellular sources. Furthermore, the impact of desflurane on the lungs after HIR extends beyond SIRT1-mediated HMGB1 trafficking regulation. Volatile anesthetics can also have antioxidant effects (Zheng et al., 2022), pulmonary endothelial glycocalyx (Casanova et al., 2016), tight junctions (Chai et al., 2015), and other signaling pathways. These uncharted mechanisms require further investigation. Finally, acute lung injury is often the initiating event for chronic complications such as pulmonary fibrosis. Our findings that desflurane limits acute HMGB1 release suggest a potential for long-term benefit, as HMGB1 is a known driver of chronic fibroproliferation (Lin et al., 2022). Future longitudinal studies are needed to evaluate the long-term benefits of desflurane in juvenile models.

## 5 Conclusion

In conclusion, the present study demonstrates that desflurane post-conditioning exerts lung protection against HIR induced ALI in young rats. The potential mechanism is primarily mediated by inhibiting HMGB1 acetylation and its subsequent nucleocytoplasmic translocation, thereby suppressing the activation of the HMGB1/TLR4/RAGE/NF-κB signaling axis in an SIRT1-dependent manner.

## Data Availability

The original contributions presented in the study are included in the article/Supplementary Material, further inquiries can be directed to the corresponding authors.

## Supplementary material

SUPPLEMENTARY FIGURE S1 Validation of antibody specificity using secondary antibody-only controls. Representative immunofluorescence images of lung tissues demonstrating the specificity of the HMGB1 (green) and TLR4 (red) antibody. The experimental group incubated with the primary target antibody exhibits distinct specific fluorescent signals. The negative control group, processed identically but incubated with an omission of the primary antibody, shows a complete absence of non-specific background fluorescence in the target channel. Cell nuclei were counterstained with DAPI (blue). Scale bar, 50 µm.

## References

[B1] Allaouchiche B. Debon R. Goudable J. Chassard D. Duflo F. (2001). Oxidative stress status during exposure to propofol, sevoflurane and desflurane. Anesth. Analg. 93, 981–985. 10.1097/00000539-200110000-00036 11574369

[B2] Boost K. A. Hofstetter C. Flondor M. Betz C. Homann M. Pfeilschifter J. (2006). Desflurane differentially affects the release of proinflammatory cytokines in plasma and bronchoalveolar fluid of endotoxemic rats. Int. J. Mol. Med. 17, 1139–1144. 10.3892/ijmm.17.6.1139 16685427

[B3] Cao Y. Zhi J. Ren H. Sheng M. Jia L. Weng Y. (2023). Association between serum HMGB1 elevation and early pediatric acute respiratory distress syndrome: a retrospective study of pediatric living donor liver transplant recipients with biliary atresia in China. BMC Anesthesiol. 23, 87. 10.1186/s12871-023-02040-0 36944948 PMC10028322

[B4] Casanova J. Simon C. Vara E. Sanchez G. Rancan L. Abubakra S. (2016). Sevoflurane anesthetic preconditioning protects the lung endothelial glycocalyx from ischemia reperfusion injury in an experimental lung autotransplant model. J. Anesth. 30, 755–762. 10.1007/s00540-016-2195-0 27255449

[B5] Chai J. Long B. Liu X. Li Y. Han N. Zhao P. (2015). Effects of sevoflurane on tight junction protein expression and PKC-α translocation after pulmonary ischemia-reperfusion injury. Exp. Mol. Med. 47, e167. 10.1038/emm.2015.27 26045255 PMC4491722

[B6] Chen R. Kang R. Tang D. (2022). The mechanism of HMGB1 secretion and release. Exp. Mol. Med. 54, 91–102. 10.1038/s12276-022-00736-w 35217834 PMC8894452

[B7] Chi X. Yao W. Zhang A. Ge M. Cai J. Zhou S. (2015). Downregulation of lung toll-like receptor 4 could effectively attenuate liver transplantation-induced pulmonary damage at the early stage of reperfusion. Mediat. Inflamm. 2015, 383907. 10.1155/2015/383907 26491225 PMC4603309

[B8] Cho Y. J. Bae J. Kim T. K. Hong D. M. Seo J. H. Bahk J. H. (2017). Microcirculation measured by vascular occlusion test during desflurane-remifentanil anesthesia is superior to that in propofol-remifentanil anesthesia in patients undergoing thoracic surgery: subgroup analysis of a prospective randomized study. J. Clin. Monit. Comput. 31, 989–997. 10.1007/s10877-016-9937-2 27672018

[B9] Figueira E. R. R. Rocha-Filho J. A. Lanchotte C. Coelho A. M. M. Nakatani M. Tatebe E. R. (2019). Sevoflurane preconditioning plus post-conditioning decreases inflammatory response with hemodynamic recovery in experimental liver ischemia reperfusion. Gastroenterol. Res. Pract. 2019, 5758984. 10.1155/2019/5758984 31093276 PMC6476030

[B10] Fujinaga T. Nakamura T. Fukuse T. Chen F. Zhang J. Ueda S. (2006). Isoflurane inhalation after circulatory arrest protects against warm ischemia reperfusion injury of the lungs. Transplantation 82, 1168–1174. 10.1097/01.tp.0000237207.73439.2e 17102768

[B11] Gao R. Ma Z. Hu Y. Chen J. Shetty S. Fu J. (2015). Sirt1 restrains lung inflammasome activation in a murine model of sepsis. Am. J. Physiol. Lung Cell Mol. Physiol. 308, L847–L853. 10.1152/ajplung.00274.2014 25659903 PMC4398874

[B12] Han D. Li X. Li S. Su T. Fan L. Fan W. S. (2017). Reduced silent information regulator 1 signaling exacerbates sepsis-induced myocardial injury and mitigates the protective effect of a liver X receptor agonist. Free Radic. Biol. Med. 113, 291–303. 10.1016/j.freeradbiomed.2017.10.005 28993270

[B13] Hassan S. Anouti A. Tan Q. Wangensteen K. Aqul A. (2025). Liver transplantation for pediatric genetic and metabolic disorders. Liver Transpl. 31, 803–814. 10.1097/LVT.0000000000000454 39171972

[B14] Hoeppner L. H. (2022). Assessing molecular regulation of vascular permeability using a VEGF-inducible zebrafish model. Methods Mol. Biol. Clift. N.J. 2475, 339–350. 10.1007/978-1-0716-2217-9_25 35451770

[B15] Hua S. Ma M. Fei X. Zhang Y. Gong F. Fang M. (2019). Glycyrrhizin attenuates hepatic ischemia-reperfusion injury by suppressing HMGB1-dependent GSDMD-Mediated kupffer cells pyroptosis. Int. Immunopharmacol. 68, 145–155. 10.1016/j.intimp.2019.01.002 30634142

[B16] Jhun J. Lee S. Kim H. Her Y. M. Byun J. K. Kim E. K. (2015). HMGB1/RAGE induces IL-17 expression to exaggerate inflammation in peripheral blood cells of hepatitis B patients. J. Transl. Med. 13, 310. 10.1186/s12967-015-0663-1 26391982 PMC4576399

[B17] Kawanishi R. Kakuta N. Sakai Y. Hari Y. Sasaki H. Sekiguchi R. (2022). Desflurane improves lung collapse more than propofol during one-lung ventilation and reduces operation time in lobectomy by video-assisted thoracic surgery: a randomized controlled trial. BMC Anesthesiol. 22, 125. 10.1186/s12871-022-01669-7 35488195 PMC9052625

[B18] Kohli R. Cortes M. Heaton N. D. Dhawan A. (2018). Liver transplantation in children: state of the art and future perspectives. Arch. Dis. Child. 103, 192–198. 10.1136/archdischild-2015-310023 28918383

[B19] Lepagnot D. (2015). Liver transplantation. Rev. l'infirmiere 64(207), 27–28. 10.1016/j.revinf.2014.10.014 26144512

[B20] Lin X. Ju Y. N. Gao W. Li D. M. Guo C. C. (2018). Desflurane attenuates ventilator-induced lung injury in rats with acute respiratory distress syndrome. Biomed. Res. Int. 2018, 7507314. 10.1155/2018/7507314 29670906 PMC5833253

[B21] Lin L. Li J. Song Q. Cheng W. Chen P. (2022). The role of HMGB1/RAGE/TLR4 signaling pathways in cigarette smoke-induced inflammation in chronic obstructive pulmonary disease. Immun. Inflamm. Dis. 10, e711. 10.1002/iid3.711 36301039 PMC9552978

[B22] Liu J. Man K. (2023). Mechanistic insight and clinical implications of ischemia/reperfusion injury post liver transplantation. Cell. Mol. Gastroenterol. Hepatol. 15, 1463–1474. 10.1016/j.jcmgh.2023.03.003 36940849 PMC10160787

[B23] Liu Y. Lu T. Zhang C. Xu J. Xue Z. Busuttil R. W. (2019). Activation of YAP attenuates hepatic damage and fibrosis in liver ischemia-reperfusion injury. J. Hepatol. 71, 719–730. 10.1016/j.jhep.2019.05.029 31201834 PMC6773499

[B24] Liu Z. Xu J. Que T. Que S. Valenti L. Zheng S. (2025). Molecular mechanisms of Ischemia/Reperfusion Injury and graft dysfunction in liver transplantation: insights from multi-omics studies in rodent animal models. Int. J. Biol. Sci. 21, 2135–2154. 10.7150/ijbs.109449 40083684 PMC11900806

[B25] Mangus R. S. Kinsella S. B. Farar D. T. Fridell J. A. Woolf L. T. Kubal C. A. (2018). Impact of volatile anesthetic agents on early clinical outcomes in liver transplantation. Transpl. Proc. 50, 1372–1377. 10.1016/j.transproceed.2018.03.001 29880359

[B26] Müller-Edenborn B. Frick R. Piegeler T. Schläpfer M. Roth-Z' graggen. B. Schlicker A. (2015). Volatile anaesthetics reduce neutrophil inflammatory response by interfering with CXC receptor-2 signaling. Br. J. Anaesth. 114, 143–149. 10.1093/bja/aeu189 24989774

[B27] Ohsumi A. Marseu K. Slinger P. McRae K. Kim H. Guan Z. (2017). Sevoflurane Attenuates ischemia-reperfusion injury in a rat lung transplantation model. Ann. Thorac. Surg. 103, 1578–1586. 10.1016/j.athoracsur.2016.10.062 28190546

[B28] O’Gara B. Talmor D. (2016). Lung protective properties of the volatile anesthetics. Intensive Care Med. 42, 1487–1489. 10.1007/s00134-016-4429-x 27376746 PMC4992441

[B29] Penkala I. J. Liberti D. C. Pankin J. Sivakumar A. Kremp M. M. Jayachandran S. (2021). Age-dependent alveolar epithelial plasticity orchestrates lung homeostasis and regeneration. Cell Stem Cell 28, 1775–1789.e5. 10.1016/j.stem.2021.04.026 33974915 PMC8500919

[B30] Prantner D. Nallar S. Vogel S. N. (2020). The role of RAGE in host pathology and crosstalk between RAGE and TLR4 in innate immune signal transduction pathways. FASEB J. 34, 15659–15674. 10.1096/fj.202002136R 33131091 PMC8121140

[B31] Rabadi M. M. Xavier S. Vasko R. Kaur K. Goligorksy M. S. Ratliff B. B. (2015). High-mobility group box 1 is a novel deacetylation target of Sirtuin1. Kidney Int. 87, 95–108. 10.1038/ki.2014.217 24940804 PMC4270955

[B32] Schilling T. Kozian A. Kretzschmar M. Huth C. Welte T. Bühling F. (2007). Effects of propofol and desflurane anaesthesia on the alveolar inflammatory response to one-lung ventilation. Br. J. Anaesth. 99, 368–375. 10.1093/bja/aem184 17621602

[B33] Schilling T. Kozian A. Senturk M. Huth C. Reinhold A. Hedenstierna G. (2011). Effects of volatile and intravenous anesthesia on the alveolar and systemic inflammatory response in thoracic surgical patients. Anesthesiology 115, 65–74. 10.1097/ALN.0b013e318214b9de 21399490

[B34] Strosing K. M. Faller S. Gyllenram V. Engelstaedter H. Buerkle H. Spassov S. (2016). Inhaled anesthetics exert different protective properties in a mouse model of ventilator-induced lung injury. Anesth. Analg. 123, 143–151. 10.1213/ANE.0000000000001296 27023766

[B35] Sucre J. Vickers K. C. Benjamin J. T. Plosa E. J. Jetter C. S. Cutrone A. (2020). Hyperoxia injury in the developing lung is mediated by mesenchymal expression of Wnt5A. Am. J. Respir. Crit. Care Med. 201, 1249–1262. 10.1164/rccm.201908-1513OC 32023086 PMC7233334

[B36] Suzuki S. Toledo-Pereyra L. H. Rodriguez F. J. Cejalvo D. (1993). Neutrophil infiltration as an important factor in liver ischemia and reperfusion injury. Modulating effects of FK506 and cyclosporine. Transplantation 55, 1265–1272. 10.1097/00007890-199306000-00011 7685932

[B37] Tsung A. Sahai R. Tanaka H. Nakao A. Fink M. P. Lotze M. T. (2005). The nuclear factor HMGB1 mediates hepatic injury after murine liver ischemia-reperfusion. J. Exp. Med. 201, 1135–1143. 10.1084/jem.20042614 15795240 PMC2213120

[B38] Ueno H. Matsuda T. Hashimoto S. Amaya F. Kitamura Y. Tanaka M. (2004). Contributions of high mobility group box protein in experimental and clinical acute lung injury. Am. J. Respir. Crit. Care Med. 170, 1310–1316. 10.1164/rccm.200402-188OC 15374839

[B39] Weber D. J. Allette Y. M. Wilkes D. S. White F. A. (2015). The HMGB1-RAGE inflammatory pathway: implications for brain injury-induced pulmonary dysfunction. Antioxid. Redox Signal. 23, 1316–1328. 10.1089/ars.2015.6299 25751601 PMC4685484

[B40] Wei S. Gao Y. Dai X. Fu W. Cai S. Fang H. (2019). SIRT1-mediated HMGB1 deacetylation suppresses sepsis-associated acute kidney injury. Am. J. Physiol. Ren. Physiol. 316, F20–F31. 10.1152/ajprenal.00119.2018 30379096

[B41] Wetterkamp M. Meiser A. Weber T. P. Vogelsang H. Lange T. Trost M. (2021). Spontaneous breathing for managing analgesia during balanced anesthesia with remifentanil and desflurane: a prospective, single center randomized controlled trial. Med. Gas. Res. 11, 94–99. 10.4103/2045-9912.310606 33942778 PMC8174411

[B42] Woo Y. D. Jeong D. Chung D. H. (2021). Development and functions of alveolar macrophages. Mol. Cells 44, 292–300. 10.14348/molcells.2021.0058 33972474 PMC8175155

[B43] Xu S. Zeng Z. Zhao M. Huang Q. Gao Y. Dai X. (2019). Evidence for SIRT1 mediated HMGB1 release from kidney cells in the early stages of hemorrhagic shock. Front. Physiol. 10, 854. 10.3389/fphys.2019.00854 31333497 PMC6625367

[B44] Yang Y. Liu Y. Wang Y. Chao Y. Zhang J. Jia Y. (2022). Regulation of SIRT1 and its roles in inflammation. Front. Immunol. 13, 831168. 10.3389/fimmu.2022.831168 35359990 PMC8962665

[B45] Yao W. Li H. Luo G. Li X. Chen C. Yuan D. (2017). SERPINB1 ameliorates acute lung injury in liver transplantation through ERK1/2-mediated STAT3-dependent HO-1 induction. Free Radic. Biol. Med. 108, 542–553. 10.1016/j.freeradbiomed.2017.04.011 28427999

[B46] Zhang W. Song J. Li W. Kong D. Liang Y. Zhao X. (2020). Salvianolic acid D alleviates cerebral ischemia-reperfusion injury by suppressing the cytoplasmic translocation and release of HMGB1-Triggered NF-κB activation to inhibit inflammatory response. Mediat. Inflamm. 2020, 9049614. 10.1155/2020/9049614 32410871 PMC7204335

[B47] Zhao H. Huang H. Ologunde R. Lloyd D. G. Watts H. Vizcaychipi M. P. (2015). Xenon treatment protects against remote lung injury after Kidney Transplantation in rats. Anesthesiology 122, 1312–1326. 10.1097/ALN.0000000000000664 25856291

[B48] Zheng Y. Lu H. Huang H. (2020). Desflurane preconditioning protects against renal ischemia-reperfusion injury and inhibits inflammation and oxidative stress in rats through regulating the Nrf2-Keap1-ARE signaling pathway. Drug design, development Therapy 14, 1351–1362. 10.2147/DDDT.S223742 32308368 PMC7138619

[B49] Zheng F. Wu X. Zhang J. Fu Z. Zhang Y. (2022). Sevoflurane reduces lipopolysaccharide-induced apoptosis and pulmonary fibrosis in the RAW264.7 cells and mice models to ameliorate acute lung injury by eliminating oxidative damages. Redox Rep. 27, 139–149. 10.1080/13510002.2022.2096339 35801580 PMC9272930

[B50] Zhou Y. Zhang F. Ding J. (2022). As a modulator, multitasking roles of SIRT1 in respiratory diseases. Immune Netw. 22, e21. 10.4110/in.2022.22.e21 35799705 PMC9250864

