# Supplementary figures and images for: Desflurane post-conditioning inhibits HMGB1 nucleocytoplasmic translocation via the SIRT1/HMGB1 axis to attenuate hepatic ischemia-reperfusion-induced acute lung injury in young rats
Source: Front Pharmacol. 2026 Jun 29;17:1847195. doi: 10.3389/fphar.2026.1847195 (PMC13357140; doi:10.3389/fphar.2026.1847195)

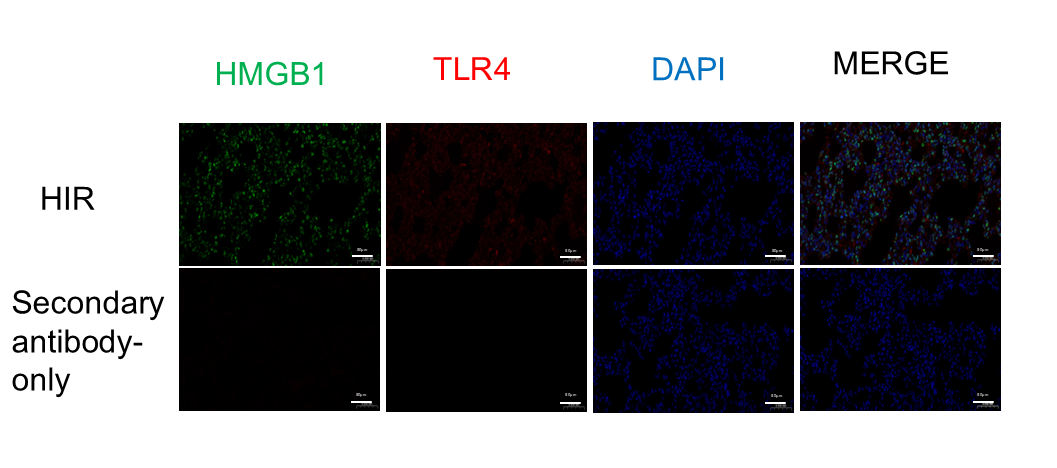

Supplement: Supplementary file 1 [file Image1.tif]
